# Size Perception of a Sport Target as a Function of Practice Success Conditions

**DOI:** 10.3389/fpsyg.2021.768131

**Published:** 2022-01-18

**Authors:** Krystina Bianchi, Molly Brillinger, Jae Todd Patterson

**Affiliations:** ^1^Department of Kinesiology, Brock University, St. Catharines, ON, Canada; ^2^Faculty of Kinesiology & Physical Education, University of Toronto, Toronto, ON, Canada

**Keywords:** motor learning, practice conditions, retention, metacognition, learner-controlled, perception

## Abstract

Superior motor task success has been correlated with participants’ self-reports of a larger-than-actual size of a sport-related target. In the present study, we examined whether a putting practice condition with greater success would differentially impact participants’ self-reported perceptions of the size of the putting hole during acquisition and retention. We randomly assigned participants to one of three different practice conditions (success-early, success-late, and self-controlled success) and had them self-report their perceived size of the putting hole upon completion of each required putting distance (25, 50, 75, 100, 125, 150, 175, 200 cm). Although there were no statistically significant differences between motor task success in the acquisition or retention period for the practice conditions, self-reported perceptions of target size were impacted by practice condition. During the acquisition period, participants in the self-controlled success and success-late conditions self-reported that the putting hole was larger than did participants in the success-early condition. In the retention period, participants in the self-controlled success condition perceived the target as larger than those in the success-early condition. These findings are the first to show that practice condition, independent of task success, differentially impacted self-reported perception of a target size.

## Introduction

Previous research has shown that game-based motor task success influenced participants’ self-reported perceptions of a sport-relevant target. For example, softball players with a higher batting average upon completion of a game perceived the ball as larger than the regulation ball size, compared to those with a lower batting average in the same game (Witt and Proffitt, 2005; Gray, 2013). Similarly, Witt et al. (2008) showed that more successful golfers perceived the putting hole as larger than less successful golfers. In these studies, participants were required to choose their perceived target size among a series of different target size options (see Witt et al., 2016 for review). Further research has shown, however, that these findings were only present when the participant provided a self-report of the perceived sport-related target size after, not before, their motor performance (Witt and Dorsch, 2009). Yet, it is currently unknown whether the structure of the practice context impacts a learner’s spatial perception of a sport-related target.

Witt et al. (2016) attributed these demonstrations of larger sport target perceptions following motor task success to an action-specific account of perception. Based on the action-specific account of perception, performers perceive the spatial layout of their environment in terms of their ability to act within this environment (see Bhalla and Proffitt, 1999; Wesp et al., 2004; Witt et al., 2016). As highlighted by Witt et al. (2016), the available visual information regarding the spatial dimensions of the sport-related target remained unchanged in these experiments. The action-specific effect has been generalized to many other tasks in which self-reported spatial perception has been influenced by the performer’s ability to perform the action (Witt et al., 2016). Differences in perceptual judgment as a function of task success have been found after a single practice session (e.g., 18 hole round of golf; Witt et al., 2008), but it remains unknown whether self-reported perceptions of a sport-related target change with either increasing or decreasing task success as a function of specific practice conditions and/or on more than 1 day of practice. Further, Witt (2020) suggested that, although perception cannot be measured directly, perceptual judgments of a sport-related target could be influenced by post-perceptual activities that could include both the processing required to plan a motor response and perceived success in performing it.

Manipulating a player’s perception of a golf hole has been shown to impact the acquisition and retention of putting (Chauvel et al., 2015). In this experiment, participants putted to a standard sized hole that was surrounded by either a series of smaller solid circles (leading to participants’ reports of a larger putting hole) or larger solid circles (leading to participants’ reports of a smaller putting hole), reproducing the Ebbinghaus illusion. Participants putting to a hole they perceived to be larger (i.e., putting to a hole surrounded by small circles) were more successful at putting in the acquisition and retention learning phases, compared to participants putting to a hole they perceived to be smaller (i.e., to a putting hole surrounded by larger circles). Participants putting to the perceptually larger target also self-reported that the target was bigger than the target described by those putting to the perceptually smaller target. These novel findings from Chauvel et al. (2015) showed that manipulating the perception of a sport-related target differentially impacted both the self-reported perception of the sport-related target and task success during retention testing. These findings have recently been replicated among children (Bahmani et al., 2017).

Many practice conditions that facilitate motor skill learning have been shown to undermine motor performance early in practice but lead to superior motor performance later in practice (Guadagnoli and Lee, 2004; Bjork, 2018), and these practice contexts have been termed cognitively effortful. Cognitive effort has been defined as the effort invested by the learner in making decisions regarding motor planning and error detection that will advantage learning (see Lee et al., 1994, for review). Providing the learner with personal control over a specific practice factor during motor skill learning may be considered cognitively effortful, based on the increased cognitive processing demands required for individualizing a practice context, compared to practice sessions in which participants are not provided control opportunities (Carter et al., 2014; Carter and Ste-Marie, 2017; see Ste-Marie et al., 2019 for review). Suggesting that providing control increases the cognitive demands of the practice condition, has been substantiated in recent research utilizing electroencephalography (EEG) data. This research has shown increased working memory activity for processing task information (i.e., frontal and pre-frontal areas, Kim et al., 2019) in participants provided control compared to not provided control over their putting distances (Jaquess et al., 2019, 2021). Examples of providing control include controlling the repetition order of a series of to-be-learned motor tasks (Titzer et al., 1993; Keetch and Lee, 2007; Wu and Magill, 2011) or controlling the features of the motor task to be either more or less complex (Andrieux et al., 2012). In a learner-controlled practice context, motor task success is often low early in practice, with more predictable and successful motor task performance occurring later in practice and in retention (see Ste-Marie et al., 2019 for review). Based on the action-specific account of perception, as task success improves over practice in a self-controlled practice context, perceptual judgments of the size of a sport-related target should increase as well.

In addition to practice conditions that heighten cognitive demands for the duration of practice, others have showed that when the learner experiences heightened cognitive demands (e.g., correcting errors) in an externally defined practice context differentially impacts motor skill learning. For example, an external facilitative practice condition that may be manipulated is predictable task success (e.g., Maxwell et al., 2001; Poolton et al., 2005). Maxwell et al. (2001) controlled the certainty of achieving putting success by either increasing (“errorful practice”) or decreasing (“errorless practice”) the certainty of task success over the duration of a practice period. Participants in the “errorful” condition started from the furthest putting distance (e.g., 200 cm), and then putted from distances that were incrementally closer (e.g., 25 cm) to the hole over the practice period, increasing task success as practice continued. Alternatively, participants in the “errorless” condition putted closest to the hole (e.g., 25 cm) initially and then incrementally increased their distance from the hole to the farthest putting distance. Maxwell et al. (2001) found superior motor performance and skill acquisition from performers who practiced in an “errorless” versus “errorful” practice condition. They suggested that practicing in an errorless condition early in skill acquisition (e.g., practicing from the 25, 50 and 75 cm distances) minimized the development of explicit corrective strategies and led to implicit rather than explicit learning. This type of practice has been shown to better enhance skill acquisition, and performance under stress (e.g., dual tasking) compared to “errorful” practice, suggesting that less cognitively effortful practice may be best.

In the present experiment, our primary purpose was to determine whether participants’ self-reported perceptions of a sport-related target would be differentially impacted by practice condition task success during the acquisition and retention of golf-putting. Our secondary purpose was to address an identified gap in Witt (2020) account of the identification of other variables that might impact judgments of a sport-related target. Our experiment differed from previous research in the following important ways. Firstly, as opposed to previous studies that relied on participants’ single-instant self-reports of the perceived target size (e.g., Witt and Proffitt, 2005; Witt et al., 2008), we had participants in the present experiment self-report their perceptions of the target size repeatedly throughout practice and prior to the retention test 1 day later. This allowed us to examine whether their self-reported perceptions of target size were differentially impacted by task success and practice context during skill acquisition and retention. Secondly, we included self-report measures prior to putting from each distance (e.g., perceived number of putts to be holed) and after completing the required putts from each distance (e.g., satisfaction with performance). Lastly, we included practice conditions that differed as a function of when participants experienced task success – early, late or as self-controlled throughout the acquisition period. These modifications extended previous research by examining whether the structure of the practice condition and its predicted impact on task success would differentially impact the judgments of participants’ perceptions of target size. Based on the action-specific account of perception and predicted task success as a function of practice condition, we hypothesized the following: (a) participants in a “success-early” condition during acquisition and retention should self-report a larger target size compared to participants in a “success-late” condition; (b) participants in a self-controlled success condition should self-report a larger target size compared to those in a “success-late” condition; and (c) metacognitive judgments should be differentially influenced by distance from the putting hole, such that the closer the distance, the greater the report of confidence, task success and satisfaction with actual performance (e.g., number of holed putts).

## Materials and Methods

### Participants

Thirty-six undergraduate students who self-reported as novice golfers, participated in this study. This sample consisted of 12 males (*M* = 21.67, *SD* = 1.11) and 24 females (*M* = 20.75, *SD* = 1.23). An *a priori* statistical power analysis was conducted using G*Power3 (Faul et al., 2007) for using two- tailed test for difference between three independent group means when assuming a medium effect size of *f* = 0.50 (Cohen, 2013), an alpha of 0.05 and statistical power of 0.80. Results showed a required total sample of 27 participants with three equal sized groups of *n* = 9. Participants were considered novice golfers if they self-reported: (a) having not played a full round of golf within the previous 12 months or (b) having played fewer than five rounds in their entire lives (Roberts and Turnbull, 2010). Both left- and right-handed individuals were equally balanced amongst the experimental groups with two left handed individuals and 10 right handed individuals in each group. Two of the left-handed participants were male and four were female. All participants were naïve to the purpose of the experiment, and each provided informed consent prior to their participation.

### Task and Apparatus

Participants performed a golf putting task using a standardized golf putter on artificial turf located on a 0.203 m high wood platform, 3.66 m in length and 1.23 m wide. Participants were required to complete a total of 160 putts – 20 putts from eight distances ranging from 25 to 200 cm, in 25 cm increments to a standardized putting hole size of 10.80 cm in diameter (Dail and Christina, 2004). The eight putting distances were identifiable to participants by a 6 cm × 6 cm taped white square. The experimenter positioned the golf ball (Top Flite XL 7000) within the marked squares before each putt.

### Measures

Motor performance was quantified by the number of holed putts from each distance during the acquisition and retention portions of the experiment (Maxwell et al., 2001; Witt and Proffitt, 2005; Witt et al., 2008). All participants self-reported their perceptions of the size of the putting hole at the following time-points: (a) before the acquisition period; (b) after the completion of 20 putts from each of the eight distances during the acquisition period; and (c) after the completion of 20 putts from each of the two distances during the retention period.

To provide their self-reported estimate of the putting hole size, participants viewed a power point slide consisting of a full green background with a single white solid circle (1.88 cm in diameter) randomly located in one of four corners (i.e., bottom left, bottom right, top left, or top right) of the screen. The starting location of the white circle varied across trials in an attempt to prevent participants from repeating a response from a previous trial based on memory recall. Participants clicked and dragged the circle, manipulating its size (i.e., enlarging and/or shrinking), to match their perception of the putting hole size. The aspect ratio of the circle was fixed which prevented participants from creating a new shape while providing their predictions. All participant’s self-reported perceptions of putting hole sizes were saved separately for analysis.

### Procedure

All participants were tested individually. Upon arrival to the laboratory, participants completed the informed consent form. They then read through a series of computerized instruction screens on a laptop computer located on an audio-visual cart (hereafter referred to as cart). The height of the cart was 1.11 m and was situated parallel to the putting green throughout the experimental procedure. Participants self-determined the amount of time they read through the instruction slides, and they were encouraged to ask questions to clarify any of the task instructions. All participants were informed that the goal of the task was to hole as many putts as possible from each of the eight putting distances.

**FIGURE 1:**
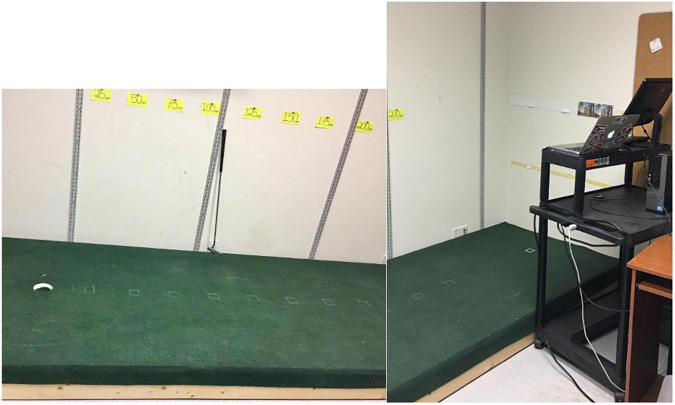
The putting green with the MacBook and Dell computer on the standing cart, located toward the back end of the putting green.

Upon completion of the instruction screens, and before any physical practice, participants provided their first self-report of their perception of the size of a regulation putting hole in Microsoft PowerPoint. Participants then proceeded to begin the physical practice portion of the acquisition phase, based on their assigned experimental condition. Throughout the experiment, when self-reporting their perceptions of the putting hole size, participants were not directly facing the putting hole. Self-reported perceptions of the putting hole size were entered on the laptop computer located on the cart, parallel to putting green, to the left of the participant. Participants were not directly facing the putting hole when providing their estimate of putting hole size (see Figure 1 for set-up). This was similar to Witt et al. (2008) method in a study showing that self-reported perceptions of a putting hole were not differentially impacted by the presence or absence of their ability to see the putting hole.

Participants were randomly assigned to one of three experimental groups: (a) the success-early group (*n* = 12), (b) the success-late group (*n* = 12) and (c) the self-controlled success group (*n* = 12). Participants assigned to the success-early group putted first from the closest distance (25 cm), progressing in 25 cm increments to the farthest distance (200 cm) from the putting hole. Participants assigned to the success-late group began at the farthest distance from the hole (200 cm) and progressed in 25 cm increments to the closest distance (25 cm). This protocol was a replication of Maxwell et al. (2001). Participants in the self-controlled success group self-selected the to-be practiced distance before each block of 20 putts, without repeating a previously practiced distance.

### Acquisition Protocol

Before putting from each distance, all participants were asked to predict how many of the 20 golf balls they believed they would hole from that distance. The purpose of this self-report measure was to determine if self-perceived putting success was differentially impacted by putting distance and/or practice condition. This measure extended previous research relating number of putts holed to task difficulty (e.g., differing putting distances) (Maxwell et al., 2001). Self-reporting perceived task success before completing the motor action was consistent with methodology in Guadagnoli and Kohl (2001). Following this estimate, participants were asked how confident they were that they could achieve their predicted success on a scale from 0 (not confident at all) to 100 (very confident) (Adkin et al., 2008). These self-reported values, as well as the actual putting score of each participant was recorded by a customized software program developed in E-Prime on a Dell computer on the cart.

To begin each block of 20 trials from each distance, and after every trial, the researcher placed a golf ball in the 6 cm × 6 cm square in the center of the putting green. A bucket of twenty golf balls were placed to the left of the putting green, beside the to-be practiced putting distance. The researcher counted the number of holed putts for each distance with a GOGO Tally Counter. Putting distances were identified using cue cards posted on the laboratory wall beside each distance. Once the twenty trials for a putting distance was complete, the experimenter removed the cue card. Thus, all participants were aware of their completed, and to-be-completed putting distances.

After completing 20 putts from each of the required distances, participants self-reported their perception of the size of the putting hole using Microsoft PowerPoint. Participants self-determined the amount of time they required to give this perception. Then, since participants were not required to count the number of putts holed at each distance, the researcher orally informed participants of the total putts holed out of 20. Participants then self-reported how satisfied they were with their performance on a scale from 0 (very dissatisfied) to 100 (very satisfied), using the customized software program in E-Prime (Adkin et al., 2008). This protocol was followed upon the completion of each putting distance (a total of eight times). The acquisition phase had a duration of approximately 60 min.

### Retention Protocol

All participants returned to the laboratory approximately 24 h after completion of their final acquisition trial. In the retention test, participants were required to perform 20 putts from the 100 cm distance followed by the 200 cm distance (Maxwell et al., 2000, 2001). The retention test protocol replicated the acquisition protocol.

### Data Analysis

To determine if there were any statistical differences between experimental groups in the participants’ initial perceptions of the putting hole size, we conducted a three group (success-early, success-late, self-controlled success) one-way ANOVA. To determine statistical differences for putting task success (i.e., number of putts holed for each distance), perceived putting hole size, confidence, satisfaction and the absolute difference between actual versus perceived putting success, between the experimental groups at acquisition, we conducted separate 3 (group: success-early, success-late, and self-controlled-success) by 8 (distance: 25, 50, 75, 100, 125, 150, 175, 200 cm) analyses of variance (ANOVA) with repeated measures on distances. Perceived golf hole size, task success, self-report measures of confidence, satisfaction, and the absolute difference between actual versus perceived putting success, between the experimental groups in the retention session was analyzed using separate 3 (group: success-early, success-late, and self-controlled success) × 2 (distance: 100 and 200 cm) ANOVAs with repeated measures on distances.

For all statistical analyses, we used *p* ≤ 0.05 as the alpha level and Tukey’s honest significant difference *post hoc* analysis to analyze any statistically significant main effects and interactions. We calculated partial eta squared (η^2^) as a measure of effect size where appropriate. We used Mauchly’s test of sphericity with the Greenhouse-Geisser adjustment when the assumption of sphericity was violated.

## Results

### Putting Success

#### Acquisition

The 3 (group: success-early, success-late, self-controlled success) × 8 (distance: 25, 50, 75, 100, 125, 150, 175, 200 cm) ANOVA with repeated measures on the last factor revealed a significant main effect for distance, *F*_(4.4,145.6)_ = 116.3, *p* < 0.001, η_p_^2^ = 0.779. Neither the main effect for group, *F*_(2,33)_ = 0.743, *p* = 0.484, nor the group × distance interaction was statistically significant, *F*_(8.8,145.6)_ = 0.956, *p* = 0.478 (see Table 1). A Tukey *post hoc* test for the distance main effect showed that the scores achieved at 25 cm were higher than those achieved at 50 to 200 cm. In summary, as the putting distance increased, the putting scores decreased, independent of experimental group.

**TABLE 1:** Participants’ acquisition means (and standard deviations) for predicted success, confidence levels, actual success, satisfaction levels and perception of putting hole size by experimental group and each putting distance.

	25 cm				
Group	Predicted	Confidence	Actual	Satisfaction	Perception
SE	17.08(1.93)	85.25(10.5)	19.92(0.3)	99.58(1.4)	11.18(2.3)
SL	19.83(0.39)	97.75(3.1)	19.92(0.3)	99.58(1.4)	12.59(1.7)
SC	17(2.18)	88.83(9.8)	20(0)	99.58(1.4)	13.5(2.1)

	**50 cm**				
**Group**	**Predicted**	**Confidence**	**Actual**	**Satisfaction**	**Perception**

SE	17.08(1.9)	86.08(7.4)	18.58(1.4)	94.92(6.7)	10.28(2.3)
SL	18.83(0.4)	94.17(4.2)	19.42(1.0)	95.58(11.6)	12.48(1.8)
SC	17(2.2)	91.42(7.2)	19.17(1.4)	96.5(6.8)	13.84(2.0)

	**75 cm**				
**Group**	**Predicted**	**Confidence**	**Actual**	**Satisfaction**	**Perception**

SE	16.5(1.9)	83.58(11.9)	18(1.6)	91.5(7.9)	10.4(1.9)
SL	17.42(2.1)	85(13.9)	18.17(1.8)	95.42(6.6)	12.53(2.0)
SC	16.17(2.5)	89.08(6.9)	17.25(2.3)	89.42(12.9)	13.88(1.7)

	**100 cm**				
**Group**	**Predicted**	**Confidence**	**Actual**	**Satisfaction**	**Perception**

SE	15.58(1.6)	84.75(8.4)	14.67(2.9)	79.58(15.1)	9.78(2.7)
SL	14.42(2.6)	80.83(11.3)	16.42(2.4)	90.5(12.3)	12.43(1.6)
SC	15.58(2.61)	88.83(10.7)	16.25(3.2)	88.92(10.8)	13.63(1.7)

	**125 cm**				
**Group**	**Predicted**	**Confidence**	**Actual**	**Satisfaction**	**Perception**

SE	13.17(2.4)	78.33(11.9)	12.5(4.1)	66.75(21.8)	9.95(2.2)
SL	13.33(2.8)	80.83(13.3)	13.58(3.7)	80.17(20.7)	12.23(1.7)
SC	13.83(3.2)	83.42(11.0)	13.83(2.6)	81.67(11.2)	13.46(2.1)

	**150 cm**				
**Group**	**Predicted**	**Confidence**	**Actual**	**Satisfaction**	**Perception**

SE	11.17(3.4)	76.67(12.9)	9.42(3.7)	53.75(22.5)	9.73(1.6)
SL	13.5(4.2)	78.67(8.7)	11.33(3.1)	70.42(22.9)	12.4(1.9)
SC	12.75(2.9)	82.83(9.2)	9.92(3.9)	55.83(29.0)	13.82(2.4)

	**175 cm**				
**Group**	**Predicted**	**Confidence**	**Actual**	**Satisfaction**	**Perception**

SE	8(3.3)	76.67(14.4)	9.33(1.9)	72.5(21.9)	9.64(2.9)
SL	9.33(2.2)	75.42(17.0)	11.08(3.0)	81.25(23.9)	12.11(1.6)
SC	10.91(4.0)	77.42(15.3)	9.83(2.1)	82.08(14.5)	13.91(2.1)

	**200 cm**				
**Group**	**Predicted**	**Confidence**	**Actual**	**Satisfaction**	**Perception**

SE	8.92(4.2)	74.17(15.8)	9.58(1.8)	66.25(21.8)	9.61(2.9)
SL	8(3.0)	75.33(21.8)	7.5(2.3)	68.33(25.3)	12.38(2.0)
SC	8(3.5)	79.92(19.0)	8.67(4.6)	67.5(32.3)	13.48(2.4)

*SE = success-early group; SL = success-late group (SL); and SC = self-control group. Acquisition consisted of eight distances (20 trials per distance).*

#### Delayed Retention Test (24-h)

The 3 (group: success-early, success-late, self-controlled success) × 2 (distance: 100, 200 cm) ANOVA with repeated measures on the last factor revealed a significant main effect for distance, *F*_(1,33)_ = 78.99, *p* < 0.001, η_p_^2^ = 0.705. Neither the main effect for group, *F*_(2,33)_ = 0.717, *p* = 0.496, nor the group × distance interaction was statistically significant, *F*_(2,33)_ = 0.887, *p* = 0.421 (see Table 2). A Tukey *post hoc* test showed task success achieved at the 100 cm distance (*M* = 15.69, *SD* = 2.79) was greater compared to the 200 cm distance (*M* = 10.47, *SD* = 3.28).

**TABLE 2:** Retention (RT) means (and standard deviations) for participants’ score predictions, confidence levels, actual putting scores, satisfaction levels and perception values by group at both distances.

	100 cm				
Group	Predicted	Confidence	Actual	Satisfaction	Perception
SE	15.25(2.3)	82.02(7.8)	15.67(2.6)	77.5(11.2)	10.66(2.3)
SL	15.42(2.6)	83.33(8.4)	15.83(2.7)	88.75(11.9)	12.5(1.8)
SC	14.08(3.6)	85.5(9.5)	14.08(3.6)	85.83(23.1)	12.99(3.1)

	**200 cm**				
**Group**	**Predicted**	**Confidence**	**Actual**	**Satisfaction**	**Perception**

SE	11.33(2.5)	74.58(11.3)	11.75(2.9)	77.08(10.3)	9.89(1.7)
SL	11.16(3.0)	74.17(15.9)	10.58(3.9)	70(25.2)	12.53(1.9)
SC	10.5(3.7)	78.67(13.8)	9.83(3.4)	74.33(22.4)	13.02(3.6)

*SE = success-early group (SE); SL = success-late group; and SC = self-control group.*

### Self-Reported Perception of Putting Hole Size

#### Pre-test

**FIGURE 2:**
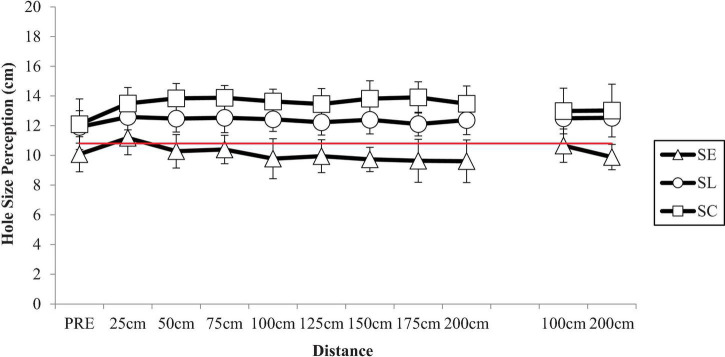
Participants’ mean self-reported hole size perceptions by experimental group at each putting distance during the acquisition phase (Day 1) and the retention phase (Day 2). Note: the red horizontal line indicates the size of the actual putting hole (10.8 cm). SE = success early; SL = success late; and SC = self-controlled success.

The one-way ANOVA revealed no statistically significant effects, *F*_(2,33)_ = 2.039, *p* = 0.146 for participants self-reported size of the putting hole before beginning physical practice in the acquisition period (see Figure 2).

#### Acquisition

The 3 (group: success-early, success-late, self-controlled success) × 8 (distance: 25, 50, 75, 100, 125, 150, 175, 200 cm) ANOVA with repeated measures on the last factor revealed a significant main effect for group, *F*_(2,33)_ = 10.988, *p* < 0.001, η_p_^2^ = 0.400. Neither the main effect for distance *F*_(2.53,83.34)_ = 2.064, *p* = 0.121, nor the group x distance interaction were statistically significant, *F*_(5.05,83.34)_ = 1.48, *p* = 0.205. A Tukey *post hoc* test showed that the self-controlled success group (*M* = 13.69, *SD* = 2.05) and the success-late group (*M* = 12.34, *SD* = 1.79) self-reported the putting hole size to be larger than the success-early group (*M* = 10.07, *SD* = 2.34). The differences between the self-controlled success group and the success-late group were not statistically significant (see Figure 2).

#### Delayed Retention (24-h)

The 3 (group: success-early, success-late, self-controlled success) × 2 (distance: 100, 200 cm) ANOVA with repeated measures on the last factor revealed a significant main effect for group on delayed retention, *F*_(2,33)_ = 4.282, *p* = 0.022, η_p_^2^ = 0.206. Neither the main effect for distance, *F*_(1,33)_ = 1.881, *p* = 0.180, nor the group × distance interaction were statistically significant, *F*_(2,33)_ = 2.374, *p* = 0.109. A Tukey *post hoc* of the group main effect showed the self-controlled success group (*M* = 13.0, *SD* = 3.31) perceived the size of the hole to be larger than the success-early group (*M* = 10.28, *SD* = 2.0), but not the success-late group (*M* = 12.52, *SD* = 1.88). The differences between the success-early and success-late group were not statistically significant (see Figure 2).

### Acquisition Metacognitive Self-Report Measures

Estimates of predicted putting success, confidence levels, actual putting success, satisfaction levels, and perception of hole size at each distance in the acquisition period can be found in Table 1.

#### Absolute Differences Between Predicted and Actual Putting Score

The 3 (group: success-early, success-late, self-controlled success) × 8 (distance: 25, 50, 75, 100, 125, 150, 175, 200 cm) ANOVA with repeated measures on the last factor revealed a significant distance × group interaction, *F*_(14,231)_ = 2.67, *p* = 0.001, η_p_^2^ = 0.139. A Tukey *post hoc* test showed that the success-early group had greater AD compared to the success-late group at 175 cm and the success-late and the self-control group at 200 cm. Further, the success-late group had significantly greater AD at 75 and 100 cm compared to 200 cm. The main effects for group (*p* = 0.221) and distance (*p* = 0.182) were not statistically significant.

#### Confidence That Predicted Success Would Match Actual Success

The 3 (group: success-early, success-late, self-controlled success) × 8 (distance: 25, 50, 75, 100, 125, 150, 175, 200 cm) ANOVA with repeated measures on the last factor revealed a significant main effect of distance *F*_(7_,_231)_ = 12.84, *p* < 0.001, η_p_^2^ = 0.280. A Tukey *post hoc* test revealed confidence levels at 25 cm were significantly greater than at 75 to 200 cm; confidence levels at 50 cm were significantly greater than at 75 to 200 cm; confidence levels at 75 cm were significantly greater than at 125 to 200 cm; confidence levels at 100 cm were significantly greater compared to 125 to 200 cm; and finally, confidence levels at 125 cm were significantly greater than at 175 to 200 cm. There main effect for group (*p* = 0.418) and the group × block interaction (*p* = 0.238) were not statistically significant.

#### Satisfaction With Task Performance at Each Distance

The 3 (group: success-early, success-late, self-controlled success) × 8 (distance: 25, 50, 75, 100, 125, 150, 175, 200 cm) ANOVA with repeated measures on the last factor revealed a significant main effect of distance *F*_(7_,_231)_ = 27.74, *p* < 0.001, η_p_^2^ = 0.457. A Tukey *post hoc* test showed satisfaction levels at 25 cm were significantly greater than at 50 to 200 cm; satisfaction levels at 50 cm were significantly greater than at 75 to 200 cm; satisfaction levels at 75 cm were significantly greater than at 100 to 200 cm; satisfaction levels at 100 cm were significantly greater than at 125 to 200 cm; satisfaction levels at 125 cm were significantly greater than at 150 cm; satisfaction levels at 150 cm were significantly greater than at 175 cm and satisfaction levels at 175 cm were significantly greater than at 200 cm. There was no significant main effect for group (*p* = 0.173), nor a group × distance interaction (*p* = 0.641).

### Retention Metacognitive Self-Report Measures

Estimates of predicted putting success, confidence levels, actual putting success, satisfaction levels, and perception of hole size at each distance in the retention period can be found in Table 2.

#### Absolute Differences Between Predicted and Actual Putting Score

The 3 (group: success-early, success-late, self-controlled success) × 2 (distance: 100, 200 cm) ANOVA with repeated measures on the last factor revealed no significant main effects for distance (*p* = 0.085) or group (*p* = 0.332), nor a group x distance interaction (*p* = 0.210).

#### Confidence That Predicted Success Would Match Actual Success

The 3 (group: success-early, success-late, self-controlled success) × 2 (distance: 100, 200 cm) ANOVA with repeated measures on the last factor revealed a significant main effect of distance *F*_(1,33)_ = 19.89, *p* < 0.001, η_p_^2^ = 0.376. A Tukey *post hoc* test showed confidence levels at 100 cm were significantly greater than at 200 cm. There was no main effect for group (*p* = 0.936), nor a group x distance interaction (*p* = 0.936).

#### Satisfaction With Task Performance at Each Distance

The 3 (group: success-early, success-late, self-controlled success) × 2 (distance: 100, 200 cm) ANOVA with repeated measures on the last factor revealed a significant main effect of distance *F*_(1,33)_ = 10.60, *p* = 0.003, η_p_^2^ = 0.243. A Tukey *post hoc* test showed satisfaction levels at 100 cm were significantly greater than at 200 cm. There was no main effect for group (*p* = 0.916), nor a group x distance interaction (*p* = 0.076).

### Self-Determined Putting Distance Order

The self-selected practice distance order for participants in the self-controlled success group can be found in Table 3. Six participants chose a “success-early” type schedule (starting at 25 cm and ending at 200 cm, consecutively), one participant chose a “success-late” type schedule (starting at 200 cm and ending at 25 cm, consecutively) and five participants chose a “random” type order of scheduling practice distances (see Table 3).

**TABLE 3:** Self-control group participants’ self-selected practice-distance order during the acquisition phase (Day 1).

Participant No.	Order	Self-Selected Schedule Type
301	25 cm, 50 cm, 75 cm, 100 cm, 125 cm, 150 cm, 175 cm, 200 cm	success-early
302	25 cm, 50 cm, 75 cm, 100 cm, 125 cm, 150 cm, 175 cm, 200 cm	success-early
303	75 cm, 125 cm, 175 cm, 200 cm, 150 cm, 100 cm, 50 cm, 25 cm	random
304	25 cm, 100 cm cm, 150 cm, 75 cm, 175 cm, 125 cm, 200 cm, 50 cm	random
305	200 cm, 175 cm, 150 cm, 125 cm, 100 cm, 75 cm, 50 cm, 25 cm	success-late
306	25 cm, 50 cm, 75 cm, 100 cm, 125 cm, 150 cm, 175 cm, 200 cm	success-early
307	25 cm, 50 cm, 75 cm, 100 cm, 125 cm, 150 cm, 175 cm, 200 cm	success-early
308	200 cm, 25 cm, 175 cm, 50 cm, 150 cm, 75 cm, 125 cm, 100 cm	Random
309	150 cm, 125 cm, 200 cm, 75 cm 175 cm, 25 cm, 50 cm, 100 cm	Random
310	25 cm, 50 cm, 75 cm, 100 cm, 125 cm, 150 cm, 175 cm, 200 cm	success-early
311	25 cm, 75 cm, 125 cm, 100 cm, 150 cm, 200 cm, 175 cm, 50 cm	Random
312	25 cm, 50 cm, 75 cm, 100 cm, 125 cm, 150 cm, 175 cm, 200 cm	success-early

## Discussion

Our primary purpose in the present experiment was to determine whether self-reported perceptions of a sport-related target would be differentially impacted by task success and various practice success conditions during motor skill acquisition and retention. Our secondary purpose was to examine whether the inclusion of additional metacognitive measures would provide further understanding of the mechanisms underlying participants’ judgments of the size of a sport-related target (see Witt, 2020). Our results from the acquisition and retention period failed to support our hypotheses (see Introduction section). In the acquisition period, participants in the self-controlled success and success-late conditions perceived the target as larger than did participants in the success-early condition. However, in the retention period, only participants in the self-controlled success condition perceived the target as larger than the success-early condition.

Based on previous research (Witt and Proffitt, 2005; Witt et al., 2008) and the action-specific account of perception, we predicted that motor task success of participants in the success-early condition in the acquisition and retention periods would result in their self-reporting a larger putting hole size compared to the participants in the success-late condition. Recall, participants in the success-late condition experienced increased task success over the duration of the acquisition period (i.e., starting at the difficult 200 cm distance and ending at the easier 25 cm distance from the hole), whereas participants in the success-early condition experienced decreasing task success as a function of increased putting distance from the hole over practice. However, our results did not support this prediction because participants in the success-late condition perceived the putting hole as larger than did the participants in the success-early condition.

We also predicted that participants in the self-controlled success condition would self-report a larger target size compared to participants in the success-late condition. We expected the self-controlled success group to report a similar (larger) target size to participants in the success-early condition during the acquisition and retention. These predictions were based on previous research showing superior learning of participants provided the opportunity to self-control the complexity of the skill acquisition environment (Wu and Magill, 2011). However, these predictions were not supported, as participants in the self-controlled success condition self-reported the putting hole as larger than participants in the success-early condition in the acquisition and retention period, and their perceptions were similar to participants in the success-late condition. When examining the structure of the self-selected putting distance order for participants in the self-controlled success condition, only one participant chose a “success-late” practice order. Other participants chose either a “random” or “success-early” type practice order. This finding is consistent with previous research that has shown that the opportunity for control, rather than the microstructure of practice, is the most important aspect of a self-controlled practice context during multi-task learning (Keetch and Lee, 2007; Wu and Magill, 2011). These results offer further insight into the impact on of a self-controlled practice context (see Ste-Marie et al., 2020 for review). Specifically, they suggest that perceptual judgments of the size of a sport-related target are also impacted in a learner-controlled practice context.

To account for these findings, we speculate that participants’ cognitive effort invested in correcting movement error and self-determining a practice distance order (self-controlled condition) influenced their judgments of putting hole size. As described earlier, cognitive effort, defined as the mental work invested by the performer in such processes as motor planning and correcting errors (Lee et al., 1994), is an important factor underlying motor skill learning (Schmidt and Bjork, 1992; Bjork, 2018). Previous research has shown that cognitive effort invested by participants during task performance increased pupil dilation (Kahneman, 1973; van der Wel and van Steenbergen, 2018 for review) and decreased heart rate variability (Patterson et al., 2016a). In the present experiment, the degree of cognitive effort invested by participants likely varied as a function of their practice condition and their subsequent task success during the acquisition period. Participants in the success-early condition experienced increased cognitive effort in correcting movement error as putting distance increased, whereas participants in the success-late condition experienced decreased cognitive effort in correcting movement error as putting distance decreased. This notion is consistent with the findings from Maxwell et al. (2001) and Poolton et al. (2005) who showed learning was a function of when participants experienced heightened demands on working memory (i.e., cognitive effort) such that practicing near the putting hole (success-early, low cognitive demand) was superior to beginning practice farthest from the putting hole (success-late practice, high cognitive demand). Differing from the success-early and success-late condition, participants in the self-controlled success condition likely experienced heightened cognitive effort throughout the acquisition period since they were deciding the order of the to-be-practiced putting distances (Wu and Magill, 2011). Our speculation is consistent with recent research examining the electroencephalography (EEG) data from participants provided versus not provided control over their putting distance from a target (Jaquess et al., 2019, 2021). Jaquess et al. (2019, 2021) showed increased neuro-cognitive engagement of working memory processing (e.g., frontal and pre-frontal areas) in learners who controlled their putting distance, compared to learners who followed an experimenter-controlled practice schedule (Jaquess et al., 2021). As noted earlier, the order of individualized practice distances for participants in the self-controlled condition was such that half of the participants chose to end practice at a distance that maximized task success (i.e., at or less than 100 cm to the target, Maxwell et al., 2001), essentially a similar experience to that of participants in the success-late condition. The other half of participants chose to end practice at the farthest distance (200 cm), similar to participants in the success-early condition. While previous research suggested that it is the opportunity to control practice, rather than the microstructure of practice that underlies the learning gains of self-controlled practice (Keetch and Lee, 2007; Wu and Magill, 2011), these data additionally suggest that experiencing cognitively demanding practice to self-determine putting distance order, and to correct movement error early (versus late) in skill acquisition leads to a self-reported larger perceived target size. Thus, our findings offer a novel insight into the potential effects of cognitive effort on perceptions of sport-related target size. However, further research is recommended to further examine the presumed underlying impact of cognitive effort on the perception of a sport-related target by using an explicit method of assessing cognitive effort (e.g., NASA TLX, see Rendell et al., 2010).

Our results for participant perceptions of target size, based on task success, were not consistent with the action-specific account of perception (Witt et al., 2016; Witt, 2020). The action-specific account predicts that task success modulates self-reported perception of a sport-related target size, and previous research has shown that participants experiencing increased task success self-reported sport-related targets as larger than participants experiencing less task success (Witt and Proffitt, 2005; Witt et al., 2008). Based on the action-specific account, the similarity of task success experienced by participants in our three experimental conditions should have resulted in relatively similar self-reports of perceived target size across these conditions. Yet, despite similar task success in these three conditions, and opposite to our prediction, our results showed that the practice conditions experienced by participants differentially impacted self-reported perceptions of target size during the acquisition and retention periods. The action specific account of perception has not been without criticism (see Firestone and Scholl, 2016; Collier and Lawson, 2018; cf. Laitin et al., 2019). Research examining specific non-perceptual factors, such as response bias of participants (e.g., transparency of the research hypothesis) have shown to challenge the predictable power of the action-specific account as a purely perceptual effect (Durgin et al., 2009; Firestone and Scholl, 2014; Collier and Lawson, 2016). Based on the findings from the present experiment, we suggest further research should continue to examine the impact of non-perceptual factors on self-reported target sizes such as participants awareness of the purposes of the experiment (e.g., response bias), perceived task demands, dissociating changes in memory versus perception of the target rather, and peripheral attentional effects, such as controlling what and for how long the participant is looking at (see Firestone and Scholl, 2016 for review). As well, further research is recommended to extend the predictions of the action-specific account by examining the impact of different practice variables on the perception of a sport-related target (e.g., practice variability, augmented feedback schedules).

To account for similarities in motor task success between practice conditions, we suggest the metacognitive processing demands (i.e., self-reporting perceived task success) experienced during the acquisition period were sufficiently influential to negate learning condition differences. Recall that before completing each putting distance, participants were required to estimate the number of putts they would hole, and their perceived confidence their actual motor task success would match their actual motor task success. Then, after completing the requisite number of putts at each distance, all participants were required to self-report their satisfaction with their performance from the just completed practice distance. The purpose of these measures was to extend previous research by examining potentially other factors contributing to judgments of perceived target size (see Witt, 2020). Previous research has shown that estimating task success (Guadagnoli and Kohl, 2001) and providing judgments of current learning (Simon and Bjork, 2001) enhanced skill acquisition compared to those not providing self-report measures. In fact, more recent research has shown that requiring participants to estimate the outcome of a just completed action, or select their likely actual outcome from a list of alternatives, resulted in similar and superior learning, compared to not estimating task success (Patterson et al., 2016b). In the present experiment, participants in the success-early condition were less accurate in estimating task success at 175 cm (compared to participants in the success-late condition) and 200 cm (compared to participants in the success-late, and self-controlled conditions) distances. However, these group differences did not result in differences in learning. Further, there was no interaction of practice condition on measures of confidence, or satisfaction. Therefore, future research should examine if engaging compared to not engaging in the metacognitive processes of a learner supersedes the impact of practice conditions modulating motor task success and self-reported perceptual judgments during motor skill acquisition. Also of importance, not finding learning advantages of a self-controlled practice context in the present experiment is not anomaly in the current research. In fact, there are other recent examples showing no learning advantages of participants provided control compared to those not provided control over aspects of their learning context (Nunes et al., 2018; Kim et al., 2019).

## Limitations and Directions for Future Research

Our findings highlighted specific methodological limitations of this study and precipitate recommendations to further understanding of the underlying mechanisms in these perceptual judgments. We required participants to estimate their perceived task success, prior to completing physical practice at a specified distance and to make perceptual judgments of the target size after completion of each of the completed putting distances. It now seems possible that engaging all participants in these preliminary judgments had a significant influence on task success, perhaps equating it to the point that the effects of learning conditions were less evident during the acquisition and retention period. In fact, previous research has shown that participants in differing practice conditions who were asked to provide a judgment of task success showed superior task success and similar learning across conditions (see Patterson et al., 2016b). To better control for these metacognitive activities, we recommend eliminating them in a future replication of the present experiment. While we used 20 learning trials at each distance, future research should also examine whether the number of practice trials at each putting distance differentially impacts judgments of target size. Another future avenue for this research is examining the perception of target size as a function of successful vs. unsuccessful putts. It is possible that perception of perceived target-size is influenced on a trial by trial basis, based on successful and unsuccessful trials. Additionally, future researchers should include the examination of different practice conditions on perception of target size (e.g., augmented feedback schedules, different types of augmented feedback) as well larger and more diverse population samples in order to determine whether results differ for older adults, elite athletes, and/or those with disabilities (e.g., persons with a cerebrovascular accident, Parkinson’s disease or Down syndrome).

## Conclusion

The results of the present experiment differed from our *a priori* hypotheses but these findings offer three novel contributions to our understanding of the factors that impact perceptions of a sport-related target. First, we found that participants who experienced increasing task success over the acquisition period (i.e., success-late practice at shorter putting distances toward the end of a practice session) and those who could control their own practice order (i.e., self-controlled success) generally perceived the sport-related target to be larger than did those who experienced decreased task success over the acquisition period (i.e., success-early practice with shorter putting distances at the beginning of a practice session). Secondly, the self-reported perception of target size was not differentially impacted by task success such that groups performed similarly in both the acquisition and retention portions of the experiment. As a result, our findings do not lend support to the action-specific account theory applied to prior findings. Rather, our results suggest that perhaps the cognitive effort invested by participants in correcting movement error, as a function of practice condition was more important than action-specific theory. Third, our use of metacognitive activities in having participants estimate their task success and express confidence in these estimates may have introduced further cognitive effort to the point that the influence on perceptual judgments from engaging in these activities may have been stronger than the influence of the learning conditions that were our primary interests. These results provide important new insights and should precipitate further study in attempts to understand the factors that can affect perceptual judgments of target size in sports research.

## Data Availability Statement

The raw data supporting the conclusions of this article will be made available by the authors, without undue reservation.

## Ethics Statement

The studies involving human participants were reviewed and approved by Social Sciences Research Ethics Board of Brock University. The patients/participants provided their written informed consent to participate in this study.

## Author Contributions

All authors listed have made a substantial, direct, and intellectual contribution to the work, and approved it for publication.

## Conflict of Interest

The authors declare that the research was conducted in the absence of any commercial or financial relationships that could be construed as a potential conflict of interest.

## Publisher’s Note

All claims expressed in this article are solely those of the authors and do not necessarily represent those of their affiliated organizations, or those of the publisher, the editors and the reviewers. Any product that may be evaluated in this article, or claim that may be made by its manufacturer, is not guaranteed or endorsed by the publisher.
